# Overcoming Multidrug Resistance by Bacterial Efflux Pump Inhibitors in Clinical *Escherichia coli* Strains

**DOI:** 10.3390/antibiotics15030276

**Published:** 2026-03-09

**Authors:** Nikoletta Szemerédi, Márta Nové, Danhui Heo, László Orosz, József Sóki, Gabriella Spengler

**Affiliations:** Department of Medical Microbiology, Albert Szent-Györgyi Health Center and Albert Szent-Györgyi Medical School, University of Szeged, Semmelweis utca 6, 6725 Szeged, Hungary; szemeredi.nikoletta@med.u-szeged.hu (N.S.); bozoki-nove.marta@med.u-szeged.hu (M.N.); heo.danhui@med.u-szeged.hu (D.H.); orosz.laszlo@med.u-szeged.hu (L.O.); soki.jozsef@med.u-szeged.hu (J.S.)

**Keywords:** multidrug resistance, *Escherichia coli*, efflux pumps, biofilm

## Abstract

**Background/Objectives**: Antimicrobial resistance (AMR) is an escalating global threat driven by antibiotic misuse and bacterial adaptation. Efflux pumps are major contributors to multidrug resistance in *Escherichia coli*, as they expel antibiotics and reduce their intracellular activity. This study examined efflux-mediated resistance in extended-spectrum beta-lactamase (ESBL)-producing *E. coli* and evaluated the potential of several efflux pump inhibitors (EPIs)—promethazine (PMZ), thioridazine (TZ), carbonyl cyanide *m*-chlorophenyl hydrazine (CCCP), reserpine (RES), and phenyl-arginine-β-naphthylamide (PAβN)—as therapeutic adjuncts. **Methods**: Antibacterial and anti-biofilm activities of EPIs were tested using broth microdilution, real-time fluorimetry, and crystal violet assays, while ceftriaxone–PMZ interactions were assessed by checkerboard analysis. **Results:** TZ and CCCP showed strain-specific antibacterial activity, whereas PMZ, RES, and PAβN did not exert any effect. PMZ, TZ, and especially CCCP effectively inhibited efflux pump function, while RES and PAβN were less active. Biofilm inhibition varied between strains, with PMZ and TZ producing moderate reductions. We observed a quite weak synergism between ciprofloxacin, ceftriaxone, and PMZ; however, the result was not significant. **Conclusions:** Overall, the results highlight the central role of efflux pumps in ESBL-producing *E. coli* and indicate that EPIs can reverse resistance (e.g., PMZ) and exhibit potent anti-biofilm activity and show additive interactions with antibiotics. However, further studies are needed to optimize their safety, pharmacokinetics, and antibiotic pairing for potential clinical use.

## 1. Introduction

Antimicrobial resistance (AMR) threatens the effectiveness of routine therapy across health-care settings [1]. Widespread antibiotic use in human medicine and agriculture, together with a limited development pipeline, has accelerated the emergence of multidrug-resistant (MDR) pathogens and complicated the management of common infections [1,2]. *Escherichia coli* is a prominent example, particularly the extended-spectrum β-lactamase (ESBL)-producing strains that compromise third-generation cephalosporins and contribute to excess morbidity in urinary, bloodstream, and postoperative infections [3,4]. These trends underscore the need for adjuvant approaches that can restore activity to existing agents rather than depending solely on new antibiotic discovery.

Among bacterial resistance mechanisms, active efflux is both pervasive and clinically significant. Efflux systems lower the intracellular drug concentrations and can lead to broad cross-resistance among different antibiotic classes. In Gram-negative bacteria, tripartite efflux pumps such as AcrAB-TolC expel β-lactams, fluoroquinolones, and many other substrates [5]. Overexpression of these pumps is a recognized cause of antibiotic failure and a frequent accompaniment of MDR phenotypes [6]. Targeting efflux thus represents a logical strategy to reverse resistance and enhance the potency of conventional antibiotics [6].

In *E. coli* specifically, two additional features further strengthen the rationale for targeting efflux mechanisms. First, efflux activity interacts with other virulence mechanisms such as biofilm formation and quorum-sensing networks, which protect bacteria from antibiotics and host defense mechanisms [7,8]. Therefore, reducing efflux activity may also disrupt biofilm development and intercellular signaling. Second, in ESBL-positive strains, treatment options are already limited, making even modest reductions in minimum inhibitory concentrations (MICs) clinically meaningful [9]. Together, these factors justify exploring efflux pump inhibitors (EPIs) as adjuncts to β-lactams and other key antibiotics in MDR *E. coli* infections, underscoring the need for careful evaluation using assays that assess both efflux function and related phenotypic outcomes.

Efflux pump inhibitors (EPIs) are molecules that inhibit efflux pumps through one or more mechanisms, ultimately leading to impaired or inactive drug transport. EPIs represent a promising resistance-modifying strategy by restoring bacterial susceptibility to antibiotics via inhibition of drug efflux systems. Their modes of action are multifaceted and include competitive or non-competitive pump inhibition, disruption of pump assembly, dissipation of the proton motive force required for active transport, modulation of efflux pump gene expression, interference with membrane components, or structural modification of antibiotics to evade pump recognition [10]. At the cellular level, these mechanisms result in increased intracellular antibiotic accumulation and prolonged target engagement, which phenotypically manifests as reduced minimum inhibitory concentrations and partial or complete restoration of antibiotic susceptibility without intrinsic bactericidal activity. From an evolutionary perspective, EPIs may reduce the selective advantage conferred by efflux-mediated resistance and potentially slow resistance development. Clinically, EPIs are therefore regarded primarily as adjuvants that potentiate existing antibiotics rather than as standalone antimicrobials, although their therapeutic application remains limited by toxicity and pharmacokinetic constraints [11,12,13]. Given this potential, EPIs have been investigated as adjuvants in antibacterial therapy. Several groups of compounds, including phenothiazines, alkaloids, peptide-based molecules, and membrane-active agents, have been shown to enhance intracellular drug retention and, in some cases, reduce the MICs of associated antibiotics [14,15,16]. However, systematic and organism-specific evaluations are still needed to determine which EPIs and inhibitor-antibiotic combinations may provide effective alternative strategies against priority pathogens such as ESBL-producing *E. coli*.

Previously, our research group investigated the activity of the AcrAB-TolC efflux pump in *E. coli* K-12 AG100 at neutral and acidic pH in the presence of the efflux pump inhibitor promethazine (PMZ). PMZ was more effective at neutral pH, while at acidic pH, it triggered a significant stress response by upregulating multiple genes. These results indicate that the regulation of the main efflux pump in *E. coli* is pH-dependent [17]. In another experiment, repurposed drugs and efflux pump inhibitors (EPIs) were evaluated against Gram-negative uropathogens to assess their antibacterial, anti-biofilm, and resistance-modifying effects under different pH conditions. Thioridazine (TZ) reduced biofilm formation and enhanced ciprofloxacin activity, while promethazine showed weaker, mostly neutral-pH effects. Other agents, including fluoxetine, sertraline, phenyl-arginine-β-naphthylamide (PAβN), carbonyl cyanide *m*-chlorophenyl hydrazone (CCCP), and V9302 ((2S)-2-amino-4-[bis[[2-[(3-methylphenyl)methoxy]phenyl]methyl]amino]butanoic acid), displayed pH-dependent antibacterial or anti-biofilm activity, indicating that repurposed drugs and EPIs could serve as adjuvants or biofilm inhibitors in treating resistant urinary tract infections [18].

In this study, we evaluated five representative EPIs, including PMZ, thioridazine, CCCP, reserpine, and PAβN, against clinical ESBL-producing *E. coli* strains. This study aimed to investigate the antibacterial properties, effects on biofilm formation, ability to inhibit efflux activity, and potential to enhance antibiotic efficacy of the selected EPIs.

## 2. Results

### 2.1. Determination of MIC

Broth microdilution assays were performed to evaluate the antibacterial properties of selected antibiotics (ampicillin, ceftriaxone, ciprofloxacin) and EPIs. For further studies, ampicillin, ceftriaxone, and ciprofloxacin were selected. All tested isolates exhibited high-level resistance to ampicillin, with MIC values of or above 128 µg/mL. Strains 131667 and 128673 showed an MIC of 128 µg/mL against ampicillin, indicating pronounced resistance. Ceftriaxone susceptibility varied markedly among the strains. Several isolates displayed elevated MIC values, including strains 131619 and 128451 (64 µg/mL) and strain 130063 (32 µg/mL). In contrast, very low MICs were observed for strains 128673 (0.008 µg/mL) and 128334 (<0.008 µg/mL), indicating high susceptibility to ceftriaxone. Intermediate MIC values (8–16 µg/mL) were detected in strains 129351, 132009, 129030, and 132014. The antibacterial activity of ciprofloxacin also demonstrated substantial heterogeneity among the strains. High-level resistance was observed in strains 131667 (128 µg/mL) and 130063 (>128 µg/mL), while elevated MICs of 32–64 µg/mL were obtained for strains 129351, 128451, 129030, and 131619. In contrast, low ciprofloxacin MICs were detected in strains 128673 (0.25 µg/mL) and strains 132014 and 132009 (2 µg/mL), suggesting preserved susceptibility in these isolates (Table 1).

In this study, five EPIs were used. These included the phenothiazine derivatives promethazine (PMZ) and thioridazine (TZ), both known to inhibit efflux pump activity and to possess potential antibacterial effects. CCCP, a protonophore, collapses the proton gradient required for efflux function, while reserpine (RES), a plant alkaloid, also acts as an efflux pump inhibitor; both are widely applied as EPIs. Additionally, PAβN, a synthetic peptidomimetic compound, is commonly used in antimicrobial research as another efflux pump inhibitor [19].

Our results demonstrated that TZ exhibited antibacterial activity against *E. coli* 129351 (MIC: 50 µg/mL), while CCCP inhibited the growth of *E. coli* 131619 (MIC: 25 µM). However, neither TZ nor CCCP displayed notable activity against the other strains tested. Similarly, PMZ, RES, and PAβN showed no detectable antibacterial effects against any of the remaining strains investigated in this study (Table 2). (For the sake of consistency and comparability, the concentration units in the table have been standardized. However, the stock concentrations of CCCP, TZ, and RES are expressed in micromolar (µM)).

### 2.2. Anti-Biofilm Activity

A biofilm inhibition assay was performed to assess the ability of various EPIs to disrupt biofilm formation. Biofilms play a critical role in bacterial survival by providing a protective niche that enhances resistance to antibiotic treatment and contributes to chronic, persistent infections.

**Figure 1:**
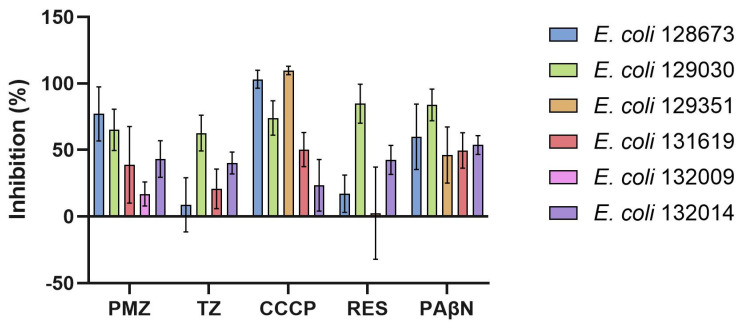
Anti-biofilm effect of EPIs on *E. coli* strains.

In this study, the impact of EPIs on biofilm formation was evaluated in ten clinical *E. coli* strains. Biofilm inhibition (%) was calculated based on mean absorbance units (AUs). Of the ten strains tested, six (1290351, 132014, 129030, 128673, 132009, and 131619) exhibited measurable anti-biofilm responses, whereas the remaining four strains (131667, 128451, 128334, and 130063) showed no inhibition. DMSO, used as a negative control, had no effect on biofilm formation. The results for the six strains demonstrating biofilm inhibition are presented in Figure 1.

### 2.3. Efflux Pump Inhibition: Ethidium-Bromide (EB) Accumulation Assay

A real-time EB accumulation assay was conducted to evaluate the efflux pump–inhibiting activity of the EPIs. EB is a common substrate of efflux pumps, enabling real-time monitoring of pump function. This assay is particularly relevant because efflux pumps significantly contribute to antibiotic resistance and biofilm formation by actively expelling antimicrobial compounds, thereby lowering their intracellular concentrations. Inhibiting these pumps can enhance antibiotic efficacy and potentially interfere with biofilm development, increasing bacterial susceptibility to treatment.

To quantify the effects of EPIs on efflux activity, the relative fluorescence index (RFI) was calculated from mean relative fluorescence unit (RFU) values. Our findings show that PMZ, TZ, and CCCP effectively inhibited efflux pump activity in all tested strains, with CCCP demonstrating the strongest inhibitory effect. In contrast, RES and PAβN did not show significant efflux pump inhibition in any of the strains examined.

**Figure 2:**
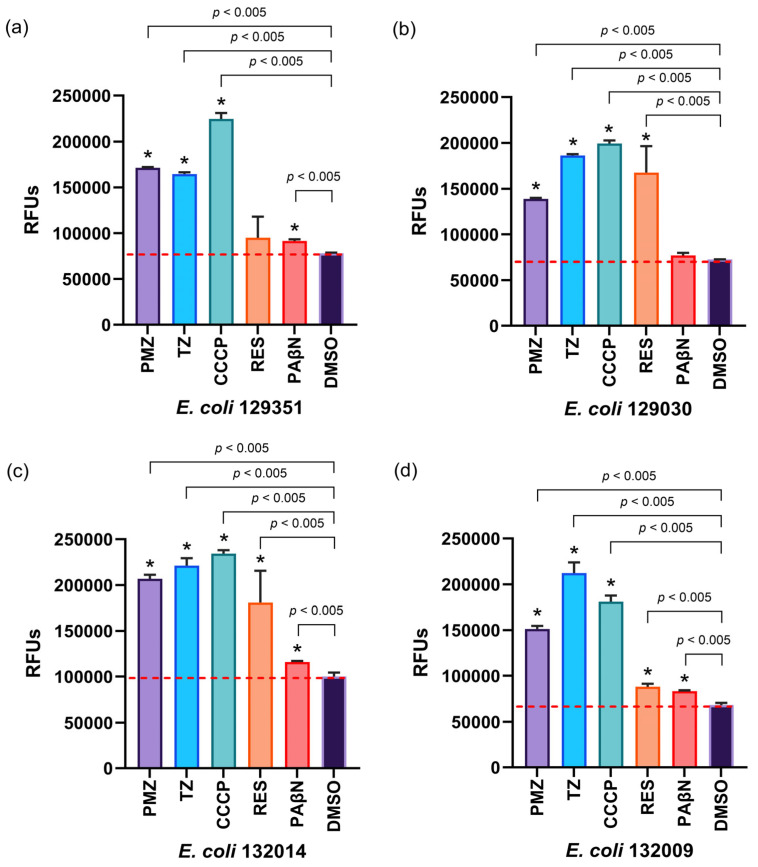
Inhibition of efflux pumps: ethidium bromide (EB) accumulation in *E. coli* strains in the presence of EPIs. The graphs show the relative fluorescence units (RFUs) of (**a**) *E. coli* 129351, (**b**) *E. coli* 129030, (**c**) *E. coli* 132014, (**d**) *E. coli* 132009 in the presence of the compounds in the 60th minute of the assay. The level of significance was * *p* < 0.005 on all strains. The red line indicates the effectiveness of the compounds compared to the solvent control.

In *E. coli* 129351, *E. coli* 129030, *E. coli* 132014, and *E. coli* 132009, the EPIs PMZ, TZ, CCCP, RES, and PAβN significantly enhanced intracellular EB accumulation, with two exceptions: RES did not increase EB accumulation in *E. coli* 129351, and PAβN showed no effect in *E. coli* 129030. DMSO was used as a negative control throughout the experiments (Figure 2).

**Figure 3:**
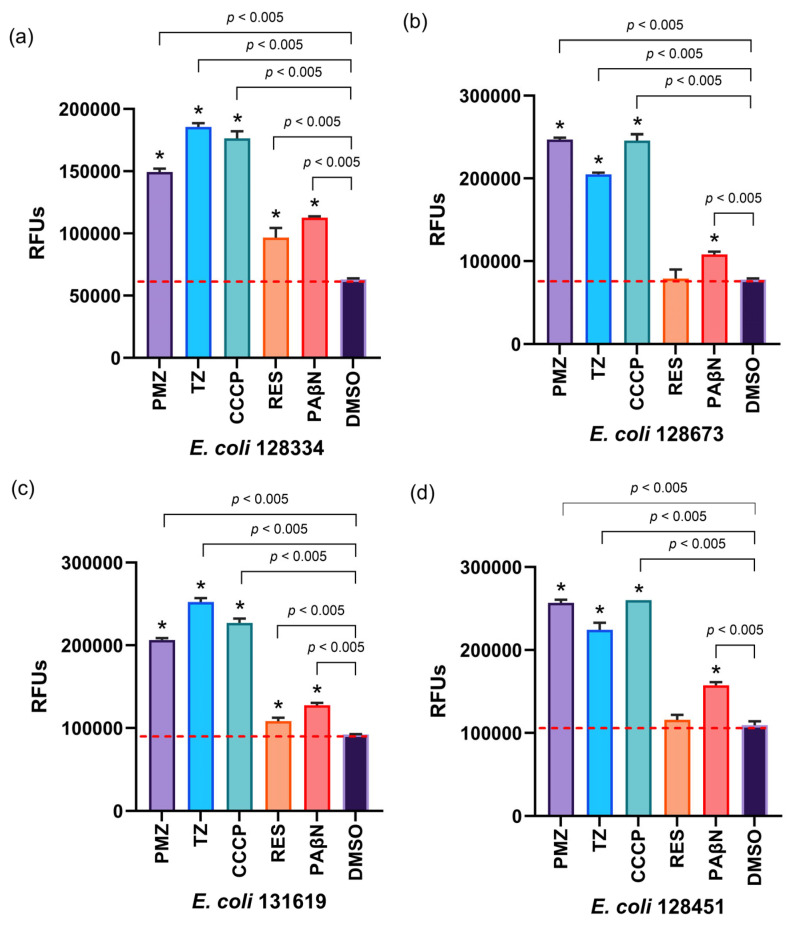
Inhibition of efflux pumps: ethidium bromide (EB) accumulation in *E. coli* strains in the presence of EPIs. The graphs show the relative fluorescence units (RFUs) of (**a**) *E. coli* 128334, (**b**) *E. coli* 128673, (**c**) *E. coli* 131619, (**d**) *E. coli* 128451 in the presence of the compounds in the 60th minute of the assay. The level of significance was * *p* < 0.005 on all strains. The red line indicates the effectiveness of the compounds compared to the solvent control. For *E. coli* 130063 and *E. coli* 131667, the EPIs PMZ, TZ, CCCP, RES, and PAβN significantly increased intracellular EB accumulation, with the exception of RES, which showed no effect in *E. coli* 131667 (Figure 4).

For *E. coli* 128334, *E. coli* 128673, *E. coli* 131619, and *E. coli* 128451, the EPIs PMZ, TZ, CCCP, RES, and PAβN significantly enhanced intracellular EB accumulation, with the exception of RES, which showed no effect in *E. coli* 128673 and *E. coli* 128451 (Figure 3).

**Figure 4:**
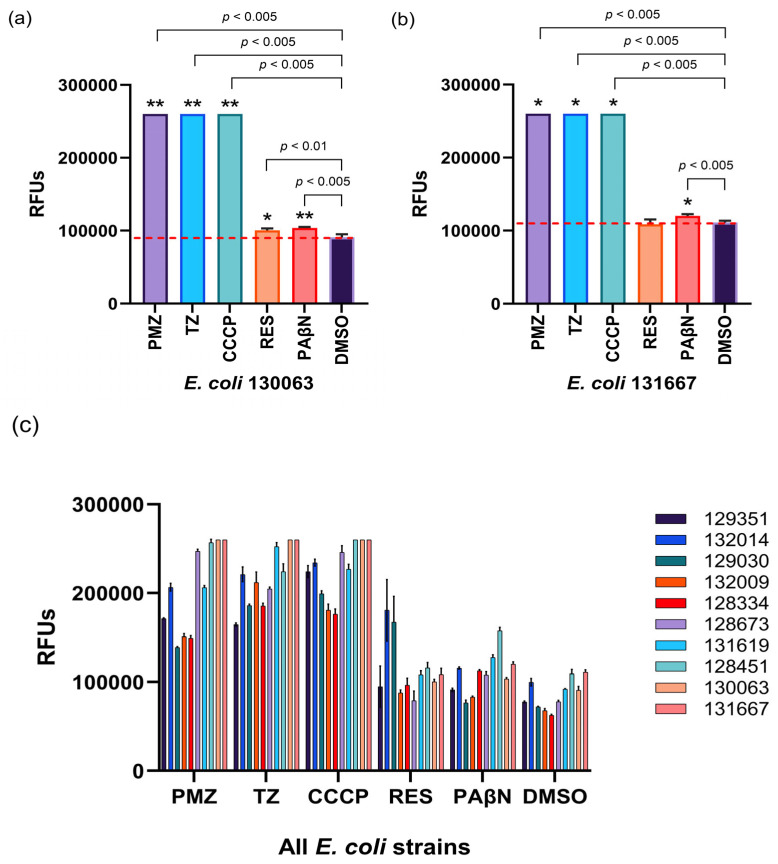
Inhibition of efflux pumps: ethidium bromide (EB) accumulation in *E. coli* strains in the presence of EPIs. The graphs show the relative fluorescence units (RFUs) of (**a**) *E. coli* 130063, (**b**) *E. coli* 131667, (**c**) all *E. coli* strains in the presence of EPIs in the 60th minute of the assay. In the case of E. coli 130063, the levels of significance were * *p* < 0.01 and ** *p* < 0.005. The level of significance was * *p* < 0.005 on *E. coli* 131667. PMZ: promethazine; TZ: thioridazine; CCCP: carbonyl cyanide m-chlorophenyl hydrazone; RES: reserpine; PAβN: phenyl-arginine-β-naphthylamide; DMSO: dimethyl sulfoxide. The red line indicates the effectiveness of the compounds compared to the solvent control.

### 2.4. Checkerboard Combination Assay

**Figure 5:**
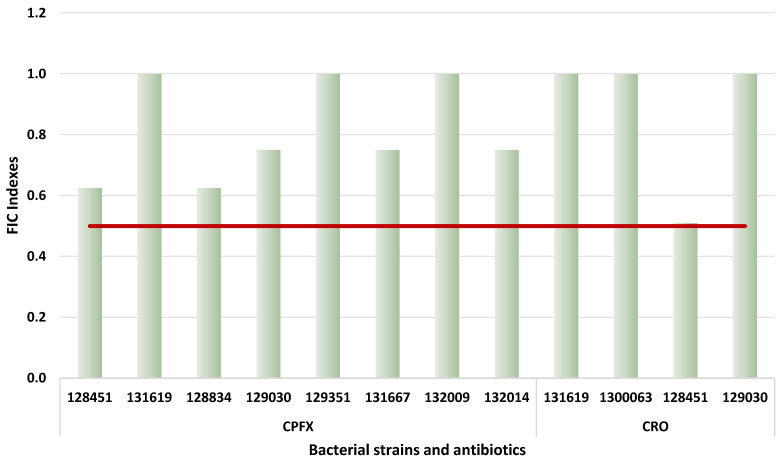
Interaction of promethazine with ciprofloxacin and ceftriaxone on *E. coli* strains (threshold is at 0.5 FIC index, indicating synergism). CPFX: ciprofloxacin; CRO: ceftriaxone. The red line indicates the threshold above which the compound is considered synergistic.

The checkerboard combination assay is widely used in vitro for assessing interactions between antimicrobial agents. In this study, PMZ, TZ, and CCCP, well-characterized EPIs frequently used in efflux pump research, were combined with ceftriaxone and ciprofloxacin to examine their interaction against *E. coli* strains through visual assessment. Ceftriaxone and ciprofloxacin were chosen based on the antibiotic susceptibility test to explore the potential synergistic effects when paired with the EPIs. The aim of this assay was to determine whether the combined use of these compounds could enhance the activity of antibiotics. For the checkerboard assay, well-defined MIC values for ceftriaxone, ciprofloxacin, TZ, CCCP, and PMZ were required for each tested strain to ensure proper plate design. The results indicated an additive interaction between promethazine and ceftriaxone, as well as between promethazine and ciprofloxacin, in all *E. coli* strains examined. A similar effect was observed in the case of CCCP and TZ combinations; however, in some instances, only an indifferent interaction was detected. When CPFX was combined with PMZ FICi of 0.625 was observed for strains 128451 and 128834. In addition, a FICi value of 0.75 was obtained for strains 131667 and 132014. For strains 131619, 129351, and 132009, the FICi was 1. In the case of the CRO and PMZ combination, the FICi value of 0.51 was detected for strain 128451; furthermore, FICi values of 1 were demonstrated for strains 131619 and 130063. However, it should be noted that in this latter case, the initial concentration of CRO was substantially higher (512 µg/mL), indicating that in vivo this combination cannot be achieved (Figure 5). PMZ was highlighted because, unlike CCCP and TZ, it has a more favorable safety profile for potential in vivo application. Images of the plates are shown in the Supplementary Material: Figures S2–S8.

### 2.5. Relative Gene Expression Analyses and Efflux Pump Inhibition in the Selected 128451 Strain

During the gene expression analysis, the expression levels of *acrA*, *acrB*, and *sdiA* were investigated, as *acrA* and *acrB* encode key components of the AcrAB-TolC multidrug efflux pump involved in antimicrobial resistance, while *sdiA* is a quorum-sensing regulator associated with bacterial stress response and biofilm formation. The effects of CPFX, PMZ, and the combination of CPFX and PMZ were evaluated. Fold change was calculated using the ΔΔCt method to quantify relative gene expression levels compared to the untreated control. Based on the obtained results, none of the treatments affected the expression of genes significantly at the applied subinhibitory concentration and exposure time, as the calculated fold change values were above 1. The CPFX+PMZ combination resulted in a fold change of 0.91 for *sdiA* (Table S3).

**Figure 6:**
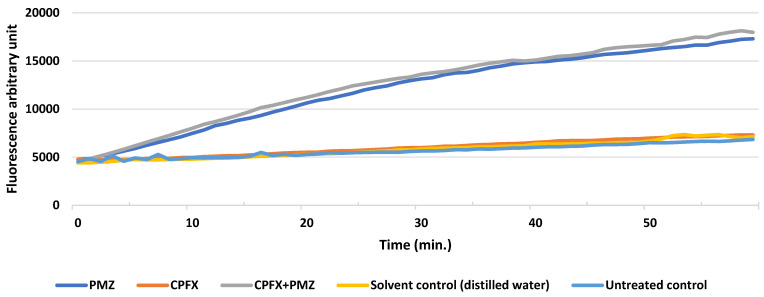
Efflux pump inhibition assessed concurrently with RNA extraction treatment. PMZ: promethazine; CPFX: ciprofloxacin.

Following one hour of treatment, an ethidium bromide accumulation assay was initiated prior to RNA extraction to assess whether the tested compounds exhibited efflux pump inhibitory activity. The compounds were applied at MIC/2 concentrations. We observed that CPFX alone did not exhibit efflux pump inhibitory activity, whereas PMZ alone, as well as the combination of PMZ and CPFX, showed inhibitory effect; however, the difference between PMZ and PMZ+CPFX was not substantial. Distilled water (DW) was used as the negative control (Figure 6).

## 3. Discussion

### 3.1. Antibacterial Activity

Antibiotic resistance continues to escalate worldwide, with multidrug-resistant (MDR) pathogens presenting significant therapeutic challenges. In recent years, numerous studies have investigated efflux pump inhibitors (EPIs) as potential tools to counteract resistance [10,20]. Earlier findings indicate that some phenothiazine derivatives—such as PMZ and TZ—possess inherent antibacterial effects against MDR bacteria like *Acinetobacter baumannii*, likely by disrupting membrane-associated energy processes and efflux activity [21]. Other EPIs, including CCCP, have been shown to compromise membrane potential and thereby improve antibiotic efficiency [10]. Conversely, compounds such as RES and PAβN typically exhibit little or no direct bactericidal activity, although they can still enhance antibiotic performance [10].

In this study, we assessed the intrinsic antibacterial properties of five EPIs against clinical ESBL-producing *E. coli* isolates. Our results showed that TZ inhibited the growth of *E. coli* 129351 (MIC: 50 µg/mL), while CCCP displayed greater potency against *E. coli* 131619 (MIC: 25 µM). PMZ, although well established as an efflux pump inhibitor, exhibited no relevant intrinsic antibacterial activity against the tested strains, with MIC values of 100 or >100 µg/mL. This observation aligns with its function as a resistance-modifying agent rather than a conventional antibacterial compound. As discussed in the literature [22], efflux pump inhibitors are primarily intended to potentiate antibiotic activity by increasing intracellular drug accumulation, rather than to exert direct bactericidal or bacteriostatic effects. In this context, minimal inherent antibacterial activity may be beneficial, as it enables modulation of resistance mechanisms while potentially limiting selective pressure on bacterial survival [22]. RES and PAβN similarly lacked measurable intrinsic antibacterial effects. The observed variability suggests that EPI-associated antibacterial activity may be strongly influenced by strain-specific traits, including efflux pump expression and membrane permeability. The absence of intrinsic antibacterial activity of a compound does not preclude its potential antimicrobial properties, such as efflux pump inhibition or interference with biofilm formation. In the present study, this distinction becomes evident: although the investigated compounds did not exhibit direct toxicity toward the bacterial strains when administered alone, they demonstrated pronounced activity in efflux pump inhibition and preventing biofilm formation.

### 3.2. Inhibition of Biofilm Formation

Biofilms play a central role in chronic infections and therapy resistance by creating a protective matrix that restricts antimicrobial access. Their formation is controlled by quorum sensing (QS) and efflux pumps, both of which promote heightened tolerance to antibiotics [23]. Previous research has shown that EPIs such as PAβN can suppress biofilm development by interfering with QS signaling and efflux pathways, weakening the bacteria’s defense mechanisms [24].

In our experiments, six of the ten *E. coli* isolates (129351, 132014, 129030, 128673, 132009 and 131619) showed marked reductions in biofilm formation after treatment with EPIs. PMZ and TZ reduced biofilm biomass by more than 50% in several strains, suggesting a potential role in diminishing biofilm-associated virulence. CCCP and PAβN also produced strong anti-biofilm effects, while RES inhibited biofilm formation more inconsistently. In contrast, four strains (131667, 128451, 128334, and 130063) did not exhibit substantial responses to any of the EPIs. These findings highlight that the anti-biofilm activity of EPIs is not universal but rather dependent on multiple strain-specific biological factors.

### 3.3. Efflux Pump Inhibition

Efflux pumps are a major driver of antibiotic resistance, as they expel antimicrobial agents from the cell and lower intracellular drug concentrations. In previous studies [25], elevated MIC values are frequently associated with the overexpression and increased activity of major RND-type efflux systems such as AcrAB-TolC, which actively reduce intracellular antibiotic concentrations. In our study, the high MIC values observed for certain antibiotics are consistent with this mechanism, as enhanced efflux activity limits effective drug accumulation within bacterial cells. This correlation supports the interpretation that efflux pump overexpression contributes significantly to the observed resistance phenotypes and underscores the relevance of efflux inhibition strategies to restore antibiotic susceptibility. Environmental pH strongly affects bacterial efflux pump activity and multidrug resistance by modulating proton motive force-dependent transport. While neutral pH generally supports optimal efflux function, pH changes encountered in host environments can influence efflux activity and increase antibiotic susceptibility, highlighting the importance of investigating pH-dependent interactions in future studies [17,26].

Compounds considered effective EPIs should meet key criteria, including broad-spectrum activity against multiple efflux pumps, clinical bioavailability without adverse effects, specific inhibition of efflux pump function, avoidance of acting as substrates for efflux pump binding sites, and the ability to prevent the development of antibiotic resistance [20]. Numerous studies have demonstrated that EPIs can restore drug susceptibility by blocking efflux activity, thereby increasing intracellular drug retention [10,20]. Because EPIs can potentiate antibiotic action, interest has grown in identifying new natural or synthetic inhibitors, including repurposed pharmaceuticals originally designed for unrelated clinical uses [27,28].

In our study, the real-time EB accumulation assay showed that PMZ, TZ, and CCCP significantly impaired efflux activity across all tested *E. coli* strains. CCCP produced the strongest response, increasing intracellular EB accumulation by as much as 2.5-fold compared to untreated controls in *E. coli* strain 129351, 129030, 130063, 131667 and 132014. PMZ and TZ also resulted in substantial increases in *E. coli* strain 132014, 132009, 128673, 128451, 130063 and 131667 (over 1.5-fold). RES and PAβN caused statistically significant changes in some cases, but these effects were considerably weaker and absent in certain strains. Overall, our findings align with previous research demonstrating that efflux inhibition varies according to the chemical structure and cellular target of the EPI, as well as bacterial adaptive mechanisms [10,20]. These results further support the importance of EPIs in restoring antibiotic activity by enhancing intracellular drug levels in MDR bacteria.

### 3.4. Checkerboard Combination Assay

The use of EPIs together with antibiotics is considered a promising approach to reverse bacterial drug resistance. Several studies have demonstrated synergistic interactions between EPIs and antibiotics, though such outcomes depend heavily on bacterial species and efflux pump expression [29,30].

In this study, we evaluated the interactions between ceftriaxone and promethazine, as well as between ciprofloxacin and promethazine, using a checkerboard assay in selected MDR *E. coli* isolates. No synergistic activity was observed for either combination in any of the tested strains; however, the types of interactions were predominantly additive. Importantly, additive effects are still considered beneficial, as they indicate that the antibiotic-EPI combinations possess enhanced antibacterial activity, potentially allowing lower effective antibiotic concentrations, reducing selective pressure for resistance development, and supporting combination therapy in strains where full synergy is not achievable. An additive effect was observed for some strains when ciprofloxacin and ceftriaxone were combined with PMZ, CCCP, and TZ. In one strain (128451), only a weak synergistic effect was observed when CRO was combined with PMZ. These findings suggest that PMZ, CCCP, and TZ generally enhance the activity of ciprofloxacin and ceftriaxone in an additive manner across the tested strains. The fact that only a single strain (128451) exhibited weak synergy with PMZ indicates that the interaction may be strain-dependent and possibly influenced by specific physiological characteristics and resistance mechanisms. Overall, while the combinations demonstrate potentiating effects, their clinical relevance may require further investigation, particularly to clarify the mechanistic basis of the observed strain-specific response.

### 3.5. Relative Gene Expression Analyses and Efflux Pump Inhibition in the Selected 128451 Strain

Although RT-qPCR revealed only minimal transcriptional changes in *acrA*, *acrB*, and *sdiA*, the compounds produced clear phenotypic effects on efflux activity and biofilm formation. CPFX alone showed no efflux pump inhibitory effect, whereas PMZ and the PMZ+CPFX combination exhibited inhibition, though the differences were not substantial. This pattern is consistent with the known mechanism of some phenothiazine type efflux pump inhibitors, which primarily act by directly interfering with pump function or membrane permeability rather than altering gene expression [31,32]. Similarly, biofilm suppression occurred without major changes in *sdiA* transcript levels, suggesting that the compounds affect biofilm formation through *sdiA*-independent pathways, such as membrane alterations, efflux inhibition, or interference with signaling processes. Functional inhibition of the AcrAB system can therefore suppress biofilm formation even in the absence of significant transcriptional changes [17,18,22].

### 3.6. Practical Use of EPIs and Current Limitations

Efflux pump inhibitors function as “helper compounds” by interfering with bacterial efflux systems that reduce intracellular antibiotic concentrations, thereby improving antibiotic efficacy. In combination therapies, these compounds can sensitize multidrug-resistant bacteria to existing antibiotics, although their clinical use is often limited by toxicity, making them valuable primarily as templates for future drug development [22]. Previous studies have highlighted that the primary value of efflux pump inhibitors lies in their ability to modulate bacterial resistance mechanisms rather than in exerting strong intrinsic antibacterial effects [22]. By inhibiting drug extrusion systems, EPIs increase intracellular antibiotic concentrations and can restore the activity of antibiotics against multidrug-resistant bacteria. Accordingly, their effectiveness is best interpreted in the context of combination therapies, where they act as adjuvants enhancing antibiotic performance rather than as standalone antimicrobial agents.

The clinical application of the EPIs used in our experiments is limited by the fact that they are toxic at the concentrations required for activity and are associated with significant toxic side effects. TZ exhibits efflux-inhibitory activity by altering membrane permeability and collapsing the proton motive force required for active efflux. Although TZ shows synergistic effects with antibiotics against multidrug-resistant bacteria, its use as an antimicrobial adjuvant is limited by severe cardiotoxic and central nervous system side effects, restricting its role to that of a lead structure for safer efflux-modulating derivatives [26,33]. Regarding TZ, originally developed as an antipsychotic, efflux inhibition was demonstrated both in vitro and in vivo against *Mycobacterium tuberculosis* [34]. CCCP is a well-established protonophore commonly used as a reference efflux inhibitor through dissipation of the proton motive force. However, its strong cytotoxicity and non-specific interference with essential cellular processes, including respiration and ATP synthesis, confine its use to in vitro mechanistic studies [35]. PAβN is a widely studied inhibitor of RND-type efflux pumps in Gram-negative bacteria, acting mainly by competitive blockage of substrate binding in transporters such as AcrAB-TolC. Nonetheless, dose-dependent toxicity and outer membrane permeabilization complicate data interpretation and have prevented its clinical development [19,36]. PMZ inhibits efflux primarily through interference with membrane-associated processes and the proton motive force. Unlike other phenothiazines, PMZ has a well-established clinical safety profile as an antihistamine [26,37] and has been safely applied as an adjuvant with gentamicin in pediatric recurrent pyelonephritis, significantly reducing infection recurrence [38]. Its weak intrinsic antibacterial activity may therefore be advantageous, supporting its role as a resistance-modifying agent rather than a standalone antimicrobial. Based on this literature data and the results of the present study, PMZ was selected for further investigation.

These early studies provide valuable insight into the feasibility of repurposing EPIs for infectious diseases. A preliminary study in India involving four patients with advanced XDR-TB found that TZ was tolerated and provided clinical improvement in two individuals, with one showing partial microbiological response. However, three patients ultimately died, likely due to the severity of their disease and the late stage of treatment [39].

Efflux pumps are key contributors to bacterial virulence and biofilm formation and are closely linked to antibiotic resistance. By exporting toxic compounds, including antibiotics and signaling molecules, they maintain cellular homeostasis and support bacterial adaptation to hostile environments. Efflux pumps modulate QS, thereby influencing biofilm development and structural stability. As a result, biofilms—multicellular communities embedded in a protective extracellular matrix—exhibit enhanced resistance to environmental stressors and host immune responses. Consequently, efflux pump inhibitors enhance antibiotic efficacy while simultaneously reducing bacterial virulence, quorum sensing, and biofilm formation [40].

Despite these encouraging results, several challenges restrict the clinical application of EPIs. Cardiotoxicity is a major concern—highlighted by the requirement for ECG monitoring during TZ therapy [39]. Additionally, optimal dosing strategies have not been standardized, and drug–drug interactions remain an issue. Future work should prioritize developing safer EPI candidates, refining dosing protocols, and reducing toxicity through systematic safety assessments. Such advancements are essential to translate EPI-based strategies into routine clinical practice.

A possible explanation for the observed effects is that the EPIs exert partial and strain-dependent inhibition of efflux pump activity, leading to a moderate increase in intracellular antibiotic accumulation rather than complete efflux blockade. This level of inhibition is sufficient to enhance antibiotic efficacy in an additive manner but may not reach the threshold required for true synergy [20]. In addition, phenothiazine derivatives such as PMZ can interfere with membrane-associated energy processes and membrane permeability, indirectly facilitating antibiotic entry [41]. Together, these mechanisms likely contribute to the observed additive interactions and anti-biofilm effects, while highlighting the complex and multifactorial nature of EPI–antibiotic interactions in MDR *E. coli*.

Furthermore, environmental factors like pH can influence bacterial efflux pump activity and thereby contribute to multidrug resistance. Acidic, neutral, and alkaline conditions modulate proton motive force-dependent efflux systems differently, affecting antibiotic accumulation, membrane permeability, and transporter efficiency. Studies show that while neutral pH often supports optimal efflux pump function, pH shifts encountered during host environments—such as gastrointestinal transit—can transiently impair efflux activity and increase bacterial susceptibility to antibiotics; therefore, it may be worthwhile to take this aspect into account and to investigate the pH-dependent interactions between these compounds and bacteria in future studies [26,42,43].

## 4. Materials and Methods

### 4.1. Compounds

Promethazine (PMZ), thioridazine (TZ), phenyl-arginine-β-naphthylamide (PAβN), reserpine (RES), carbonyl cyanide *m*-chlorophenyl hydrazone (CCCP), dimethyl sulfoxide (DMSO). The compounds were purchased from Sigma-Aldrich (St. Louis, MO, USA).

### 4.2. Reagents and Media

DMSO, phosphate-buffered saline (PBS; pH 7.4), Mueller–Hinton (MH) broth, Luria–Bertani broth (LBB) and Luria–Bertani agar (LBA) were purchased from Sigma-Aldrich (St. Louis, MO, USA). Ethidium-bromide (EB), crystal-violet (CV), and ethanol were purchased from Sigma-Aldrich Chemie GmbH (Steinheim, Germany).

### 4.3. Bacterial Strains

*E. coli* clinical strains were used in the study: *E. coli* 128334, *E. coli* 128451, *E. coli* 128673, *E. coli* 129030, *E. coli* 129351, *E. coli* 130063, *E. coli* 131619, *E. coli* 131667, *E. coli* 132009, and *E. coli* 132014. The strains were collected and provided by Dr. László Orosz (Department of Medical Microbiology, Albert Szent-Györgyi Medical School, University of Szeged, Szeged, Hungary). The antibiotic resistance characteristics of the strains are shown in Table 3.

### 4.4. Determination of Minimum Inhibitory Concentrations (MIC) by Microdilution Method

The minimum inhibitory concentrations (MICs) of both efflux pump inhibitors (EPIs) and antibiotics were determined following the protocols outlined by the Clinical and Laboratory Standards Institute (CLSI) [44]. Antibacterial efficacy was measured using a two-fold serial dilution assay performed in 96-well flat-bottom microtiter plates. Stock solutions of the EPI compounds were prepared in DMSO at initial concentrations of either 100 µg/mL or 100 µM, while antibiotics were prepared at a starting concentration of 128 µg/mL. Serial two-fold dilutions were prepared across columns 3 through 12 of the plates using 100 μL of growth medium per dilution step. Subsequently, 100 μL of a bacterial suspension—prepared as a 10^−4^ dilution of an overnight culture—was dispensed into each well except for those designated as medium controls, bringing the total volume in each well to 200 μL. The microplates were then incubated at 37 °C for 18 h. MIC values were established by visually assessing bacterial growth. DMSO was included as a negative control to verify that the solvent had no intrinsic antimicrobial effect. Experiments were conducted at neutral pH.

### 4.5. Anti-Biofilm Activity

Biofilm formation by the strains was evaluated in 96-well microtiter plates across three independent experiments using LB broth supplemented with the tested compounds. Overnight cultures were adjusted to an optical density (OD_600_) of 0.1 and dispensed into the wells, except for those designated as medium controls. The compounds were added at concentrations corresponding to ½ and ⅓ of their MIC values (Table S2), resulting in a final volume of 200 μL per well. The plates were incubated at 30 °C for 48 h with gentle shaking at 100 rpm. After incubation, the medium was discarded, and the wells were rinsed with tap water to remove non-adherent cells. Crystal violet (CV; 0.1% *w/v*) was added at 200 μL per well and allowed to stain the biofilms at room temperature for 15 min. The dye was then removed, and the plates were washed again with tap water. To quantify biofilm biomass, 200 μL of 70% ethanol was added to solubilize the bound CV, and the absorbance was measured at 600 nm using a Multiscan EX ELISA reader (Thermo Labsystems, Cheshire, WA, USA). The anti-biofilm effect of the compounds was expressed as the percentage reduction in biofilm formation relative to untreated controls. Experiments were conducted at neutral pH.

### 4.6. Real-Time Ethidium Bromide (EB) Accumulation Assay

The efflux pump–inhibitory effects of the EPIs were assessed with a real-time ethidium bromide (EB) accumulation assay, which monitors the intracellular build-up of the fluorescent efflux substrate EB. Fluorescence emitted by internalized EB was measured using a CLARIOstar Plus plate reader (BMG Labtech GmbH, Ortenberg, Germany). Bacterial cultures were grown at 37 °C with shaking until they reached an OD_600_ of 0.6, after which they were washed three times with PBS, centrifuged, and resuspended in fresh PBS. The EPI solutions were prepared at concentrations corresponding to ½ and ⅓ of their MICs (Table S2) and combined with PBS containing a non-inhibitory concentration of EB (2 µg/mL) [45]. These mixtures were dispensed into a black 96-well microtiter plate (Greiner Bio-One, Hungary), followed by the addition of 50 µL of the bacterial suspension (OD_600_ = 0.6) to each well. The plate was then placed into the CLARIOstar instrument, and fluorescence was recorded every minute over a 60 min period using excitation and emission wavelengths of 530 nm and 600 nm, respectively. Based on the resulting time-course data, the relative fluorescence index (RFI) at the final measurement point (minute 60) was calculated according to the following formula:

RFI = (RF_treated_ − RF_untreated_)/RF_untreated_

where RF_treated_ represents the relative fluorescence (RF) at the final time point of the EB retention curve in the presence of an inhibitor, and RF_untreated_ corresponds to the RF at the final time point of the EB retention curve for the untreated control containing the solvent (DMSO). Experiments were conducted at neutral pH.

### 4.7. Checkerboard Combination Assay

A checkerboard microdilution assay was employed to investigate potential synergistic effects among the EPI, promethazine, and the antibiotics ceftriaxone and ciprofloxacin against ESBL-producing *E. coli* strains and one non-ESBL strain. A schematic layout of the checkerboard plate is presented in Figure S1.

For the checkerboard assay, ceftriaxone and ciprofloxacin were subjected to two-fold serial dilutions using 100 μL per well along the horizontal axis of the microtiter plate. PMZ, CCCP, and TZ were diluted separately in tubes to a final volume of 600 μL, after which 50 μL of each two-fold dilution was dispensed down the vertical columns of the plate. The starting concentrations are shown in Table S1. It is important to note that determining the exact MIC values of the tested compounds is essential for performing checkerboard combination assays, as these values are required to properly set the experimental conditions and concentration ranges. Overnight bacterial culture was diluted in Mueller Hinton (MH) medium, and 50 μL of the bacterial suspension was added to each well. The plates were then incubated at 37 °C for 18 h. After incubation, bacterial growth was evaluated visually.

The interaction between antibiotics and PMZ/CCCP/TZ was interpreted using synergy analysis, comparing the activity of the drug combination to its individual effects. This assessment was quantified using the Fractional Inhibitory Concentration Index (FICi), which measures how much the MIC of each agent changes when used together. The FIC index is calculated using the combination that shows the greatest deviation from the standalone MIC values. The interactions between the tested compounds were determined according to the following formula:

$$\frac{A}{MICA}+\frac{B}{MICB}=FICA+FICB=FIC\;Index$$

where A and B are the MIC of each compound in combination (in a single well), and MICA and MICB are the MIC of each drug individually [46].

The calculated FIC index is then used to classify the interaction between the two tested compounds. These interactions fall into three main categories: synergistic, additive/indifferent, or antagonistic. A synergistic effect is indicated by an FIC index below 0.5, reflecting an enhanced inhibitory action in which the MICs of one or both agents decrease when used together. An additive or indifferent interaction is defined by an FIC index between 0.5 and 4, signifying either no meaningful improvement or only a slight enhancement in activity compared with the individual compounds. Antagonism is identified when the FIC exceeds 4, demonstrating that the combination results in increased MIC values or diminished overall inhibitory capacity (Table 4) [46]. Experiments were conducted at neutral pH.

### 4.8. Bacterial RNA Purification

*E. coli* 128451 strain was cultured overnight in LB broth at 37 °C with shaking (OD600: 0.6). Bacterial suspensions were treated with CPFX alone (at 32 µg/mL), PMZ alone (at 100 µg/mL) and CPFX and PMZ in combination (32 µg/mL and 100 µg/mL) in LB medium and incubated at 37 °C with shaking. The total RNA was isolated after one hour of culturing. RNA preparation was carried out in an RNase-free environment using the NucleoSpin RNA kit (Macherey Nagel, Düren, Germany) according to the manufacturer’s instructions. Purified RNA was stored in RNase-free water in nuclease-free collection tubes and was maintained at −20 °C until quantification was performed. The concentration of the extracted RNA templates was assessed by spectrophotometry at 260 nm (Bio-Rad, Hercules, CA, USA, SmartSpec™ Plus).

### 4.9. Relative Gene Expression Analyses by Real-Time Reverse Transcriptase Quantitative Polymerase Chain Reaction (RT-qPCR)

The relative gene expression levels were determined in the *E. coli* strain 128451 treated with CPFX alone, PMZ alone and CPFX and PMZ in combination. The applied concentrations of the compounds were MIC/2. The relative expression levels of *acrA*, *acrB* and *sdiA* genes were determined by RT-qPCR. This involved the CFX96 Touch Real-Time PCR Detection System (BioRad, Hercules, CA, USA), strictly following the manufacturer’s recommendations for the SensiFAST^TM^ SYBR^®^ No-ROX One-Step Kit (Bioline GmbH, Luckenwalde, Germany). Briefly, each well of the 96-well microtiter plate contained 20 µL as follows: 10 µL of the 2× SensiFAST^TM^ SYBR^®^ No-ROX One-Step Mix, 0.2 µL Reverse Transcriptase, 0.4 µL RiboSafe RNase Inhibitor, 5.4 µL Diethylpyrocarbonate (DEPC)-treated water, 500 nM of each primer and approximately 20 ng of total RNA in RNase-free water. Thermal cycling was initiated with a denaturation step of 5 min at 95 °C, followed by 40 cycles each of 10 s at 95 °C, 30 s at 57 °C, and 20 s at 72 °C. The relative quantities of the mRNA of each gene of interest were determined by the ΔΔCT method. Gene transcript levels were normalized against the *E. coli* housekeeping gene GAPDH measured in the same sample. The primers used in the assay are shown in Table 5 [45].

## 5. Conclusions

Overall, this work contributes to a better understanding of how efflux pump inhibitors (EPIs) can support efforts to overcome antibiotic resistance. Although certain EPIs displayed limited direct antibacterial activity, their strongest effects were observed in inhibiting efflux pumps and reducing biofilm formation. These results underscore the ability of EPIs to enhance the effectiveness of existing antibiotics, while also indicating that more research is needed to determine which antibiotic–EPI combinations are most suitable for therapeutic use. The notable strain-dependent differences in both biofilm suppression and efflux inhibition further suggest that individualized or targeted strategies may be necessary for optimal treatment outcomes. Future investigations should prioritize detailed pharmacokinetic assessment and safety evaluation of EPIs to advance their development as adjunct agents against MDR bacterial infections.

## Figures and Tables

**Table 1:** MIC values of the selected antibiotics against the examined *E. coli* strains.

Strains	MIC (µg/mL)		
	Ampicillin	Ceftriaxone	Ciprofloxacin
*E. coli* 129351	>128	8	32
*E. coli* 132014	>128	16	2
*E. coli* 131619	>128	64	64
*E. coli* 128451	>128	64	32
*E. coli* 132009	>128	16	2
*E. coli* 129030	>128	8	32
*E. coli* 131667	128	<0.25	128
*E. coli* 128673	128	0.008	0.25
*E. coli* 130063	>128	32	>128
*E. coli* 128334	>128	<0.008	16

**Table 2:** Minimum inhibitory concentrations (MICs) of the selected compounds for the investigated *E. coli* strains.

Strains	MIC					
	(µg/mL)		(µM)			(*v/v*%)
	PMZ	TZ	CCCP	RES	PAβN	DMSO
*E. coli* 129351	100	50	20.46	>60.87	>40.58	>2
*E. coli* 132014	100	100	20.46	>60.87	>40.58	>2
*E. coli* 131619	100	>100	5.12	>60.87	>40.58	>2
*E. coli* 128451	100	100	20.46	>60.87	>40.58	>2
*E. coli* 132009	100	>100	20.46	>60.87	>40.58	>2
*E. coli* 129030	100	100	20.46	>60.87	>40.58	>2
*E. coli* 131667	100	>100	20.46	>60.87	>40.58	>2
*E. coli* 128673	>100	100	20.46	>60.87	>40.58	>2
*E. coli* 130063	>100	>100	>20.46	>60.87	>40.58	>2
*E. coli* 128334	100	>100	20.46	>60.87	>40.58	>2

**Table 3:** The resistance profile of the examined *E. coli* strains (S: sensitive; R: resistant; ESBL: extended-spectrum beta-lactamase producing strain).

**Antibiotics**	**Identifier of *E. coli* Strains**									
	**128673**	**128451**	**131619**	**131667**	**132009**	**130063**	**132014**	**128334**	**129351**	**129030**
Ampicillin	R	R	R	R	R	R	R	R	R	R
Amoxicillin-clavulanic acid	R	R	R	R	R	R	R	S	R	R
Piperacillin-tazobactam		R			R		R		R	R
Cefotaxim		R			R		R		R	R
Sumetrolim	R	R	R	R	R	R	R	S	S	S
Cefixim	R	R	R	R	R	R	R		R	R
Ceftazidim	R	R	R	R	R	R	R		R	R
Ceftriaxon	R	R	R	R	R	R	R		R	R
Cefepim		R			R		R		R	R
Cefuroxim	R	R	R	R	R	R	R	S	R	R
Amikacin		S			S		S		S	S
Gentamycin	S	S	S	R	S	R	S		S	S
Tobramycin		S			S		S		S	S
Meropenem	S	S	S	S	S	S	S		S	S
Imipenem	S	S	S	S	S	S	S		S	S
Ciprofloxacin	S	R	R	R	R	R	R	R	R	R
Levofloxacin		R			R		R		R	R
Moxifloxacin		R			R		R		R	R
Norfloxacin	S		R	R		R				
Fosfomycin			S	S		S				
Nitrofurantoin	S		S	R		R				
Ertapenem	S	S	S	S	S	S	S		S	S
ESBL	YES	YES	YES	YES	YES	YES	YES	NO	YES	YES

**Table 4:** Checkerboard interaction types.

Types of Interaction	FICi Value
Synergy	≤0.5
Additive effect	0.5< and ≤1
Indifference	1< and ≤4
Antagonism	4<

**Table 5:** Primer sequences of the genes analyzed [45].

Genes	Forward Primer Sequences	Reverse Primer Sequences
*acrA*	CTTAGCCCTAACAGGATGTG	TTGAAATTACGCTTCAGGAT
*acrB*	CGTACACAGAAAGTGCTCAA	CGCTTCAACTTTGTTTTCTT
*sdiA*	CTGATGGCTCTGATGCGTTTA	TCTGGTGGAAATTGACCGTATT
*GAPDH*	ACTTACGAGCAGATCAAAGC	AGTTTCACGAAGTTGTCGTT

## Data Availability

This data presented in this study is available on request from the corresponding author.

## Supplementary Materials

The following supporting information can be downloaded at: https://www.mdpi.com/article/10.3390/antibiotics15030276/s1, Figure S1: Checkerboard combination plate layout (Figures S2–S4); Figure S2: Images of the combination assay: ciprofloxacin and promethazine; Figure S3: Images of the combination assay: ceftriaxone and promethazine; Figure S4: Interaction of promethazine with ceftriaxone on *E. coli* strains; Figure S5: Interaction of CCCP with ceftriaxone on *E. coli* strains, Figure S6: Interaction of CCCP with ciprofloxacin on *E. coli* strains; Figure S7: Interaction of TZ with ceftriaxon on *E. coli* strains; Figure S8: Interaction of TZ with ciprofloxacin on *E. coli* strains; Table S1: Selected starting concentrations of the tested antibiotics with PMZ, TZ and CCCP; Table S2: Summary table of the starting concentrations used for the investigation of anti-biofilm activity and efflux pump inhibitory effects; Table S3: Effects of CPFX and PMZ on gene expression (fold change).

## Abbreviations

The following abbreviations are used in this manuscript:

CPFX	Ciprofloxacin
CRO	Ceftriaxone
PMZ	Promethazine
TZ	Thioridazine
CCCP	Carbonyl cyanide m-chlorophenyl hydrazone
PAβN	Phenyl-arginine-β-naphthylamide
DMSO	Dimethyl sulfoxide
