# Supplementary material for: Overcoming Multidrug Resistance by Bacterial Efflux Pump Inhibitors in Clinical Escherichia coli Strains
Source: Antibiotics (Basel). 2026 Mar 9;15(3):276. doi: 10.3390/antibiotics15030276 (PMC13024732; doi:10.3390/antibiotics15030276)
Supplement: Supplementary file 1 [file antibiotics-15-00276-s001.zip › antibiotics-4098548-supplementary.pdf]

Supplementary information

Overcoming multidrug resistance by bacterial efflux pump inhibitors in clinical *Escherichia coli* strains

Nikoletta Szemerédi [†], Márta Nové [†], Danhui Heo [1], László Orosz [1], József Sóki [1] and Gabriella Spengler [1,*]

[1] Department of Medical Microbiology, Albert Szent-Györgyi Health Center and Albert Szent-Györgyi Medical School, University of Szeged, Semmelweis utca 6, 6725 Szeged, Hungary; szemeredi.nikoletta@med.u-szeged.hu; bozoki-nove.marta@med.u-szeged.hu; gjeksgnl123@gmail.com; orosz.laszlo@med.u-szeged.hu; soki.jozsef@med.u-szeged.hu; spengler.gabriella@med.u-szeged.hu

* Correspondence: spengler.gabriella@med.u-szeged.hu
† These authors contributed equally to this work.

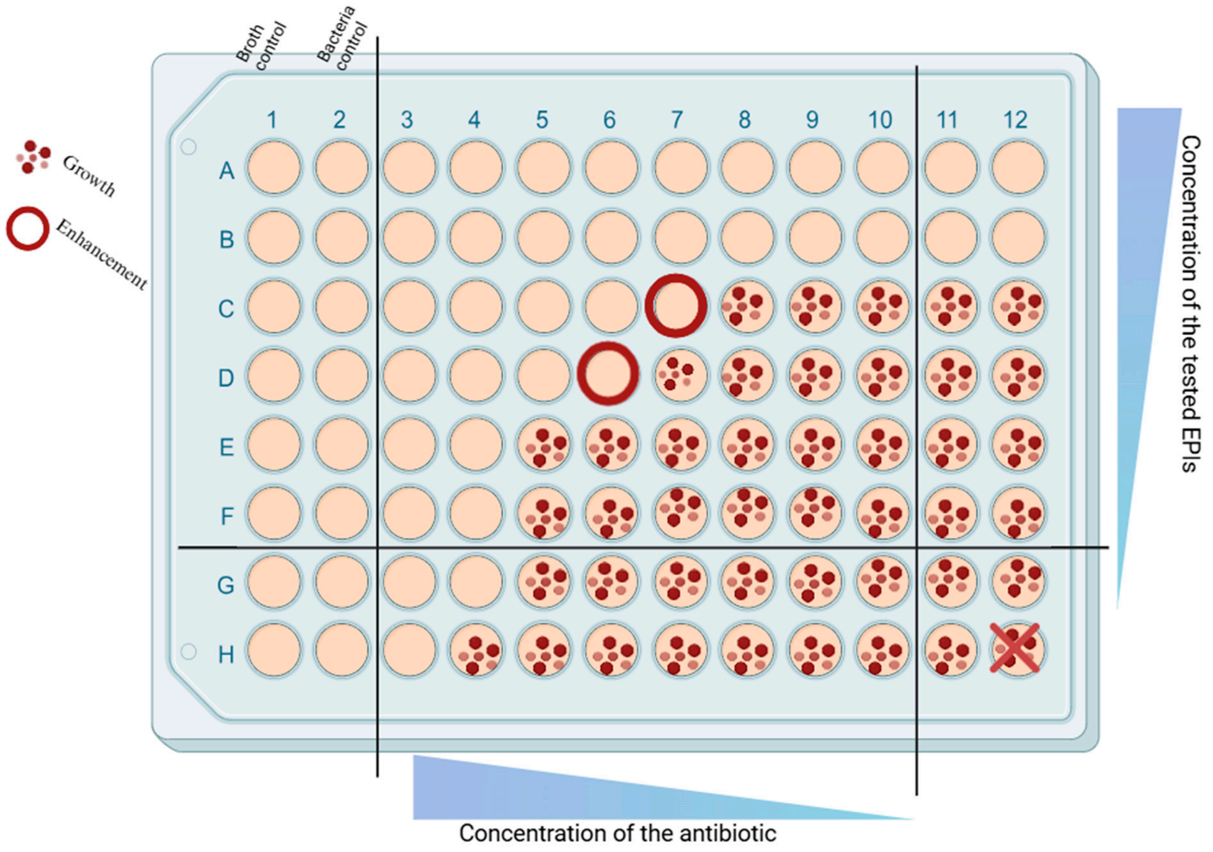

Figure S1. Checkerboard combination plate layout (Figure S2, S3 and S4).

Table S1. Selected starting concentrations of the tested antibiotics with PMZ (µg/mL), TZ (µg/mL) and CCCP (µM).

CRO: ceftriaxone, CPFX: ciprofloxacin

Identifier of selected strains	TZ	CRO	CPFX	PMZ	CCCP
**128334**	–	–	64	–	250
**128451**	250	1024	128	200	250
**128452**	–	128	–	200	–
**128673**	250	0.128	1	–	250
**128834**	–	–	128	200	–
**129030**	250	1024	128	200	250
**129351**	150	1024	128	200	250
**130063**	–	128	–	–	–
**131619**	–	1024	256	200	100
**131667**	–	–	512	200	–
**132009**	–	1024	8	200	250
**132014**	250	1024	8	200	250

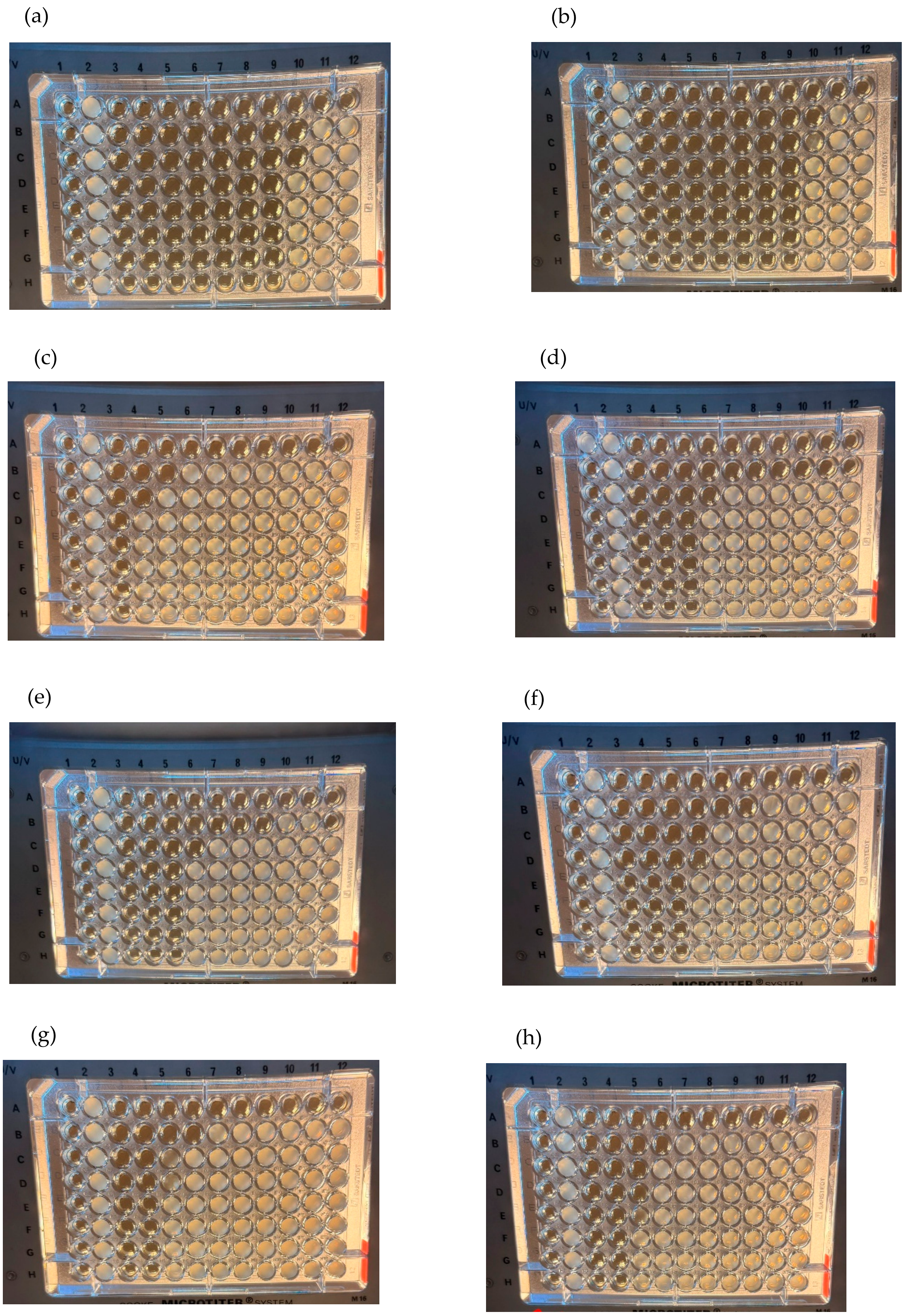

Figure S2. Images of the combination assay: ciprofloxacin and promethazine

(a) *E. coli* 132014; (b) *E. coli* 132009; (c) *E. coli* 131667; (d) *E. coli* 129351; (e) *E. coli* 129030; (f) *E. coli* 128834; (g) *E. coli* 131619; (h) *E. coli* 128451

(a)

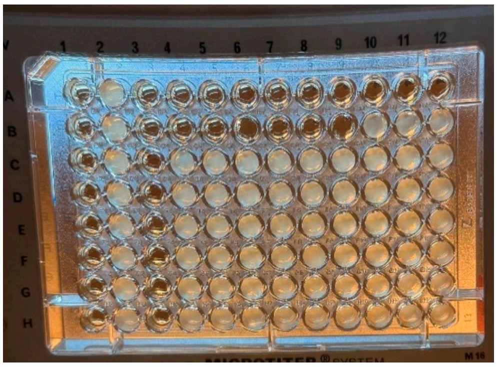

(b)

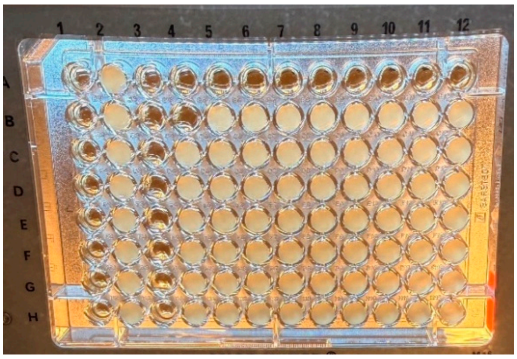

(c)

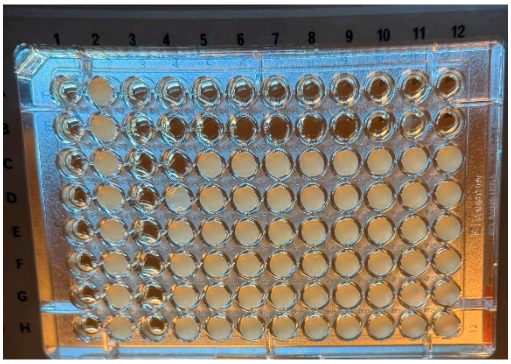

Figure S3. Pictures of the combination assay: ceftriaxone and promethazine

(a) *E. coli* 128451; (b) *E. coli* 130063; (c) *E. coli* 131619

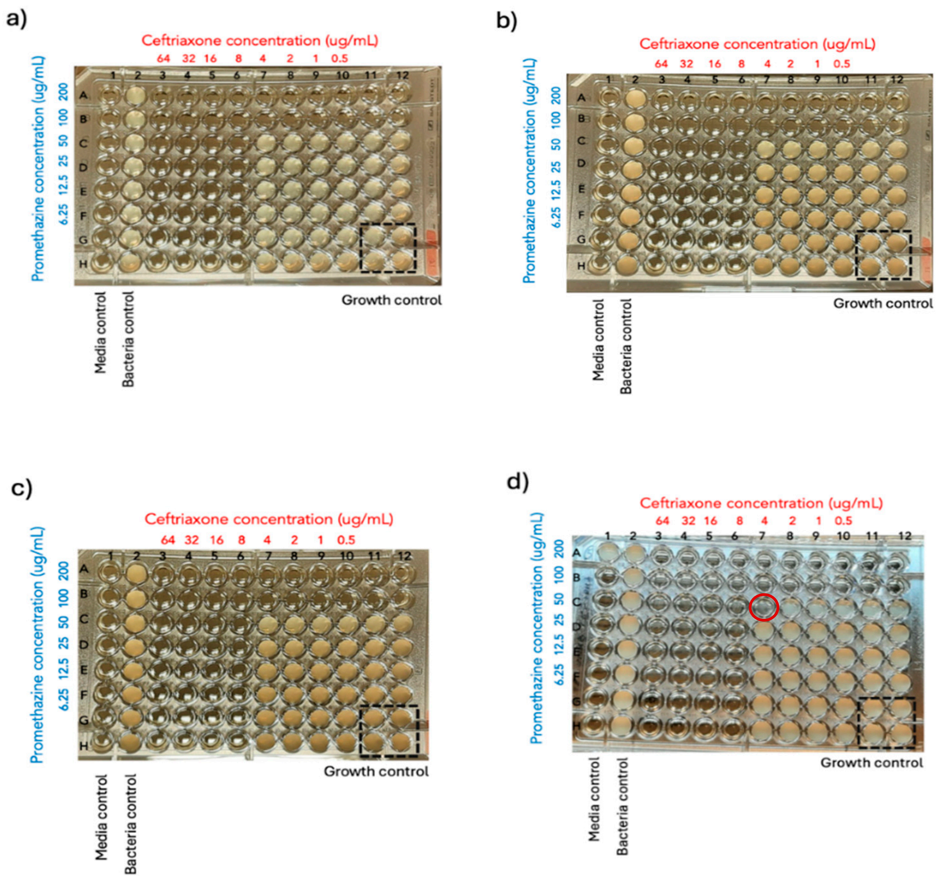

Figure S4. Interaction of promethazine with ceftriaxone on *E. coli* strains

(a) *E. coli* 129351, (b) *E. coli* 132014, (c) *E. coli* 132009, (d) *E. coli* 129030

(a)

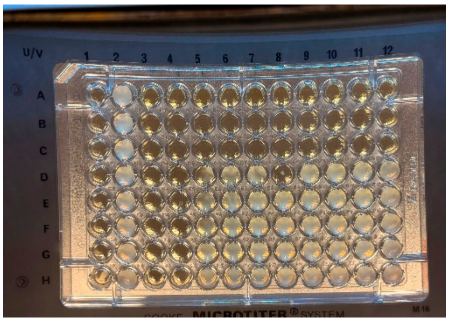

(b)

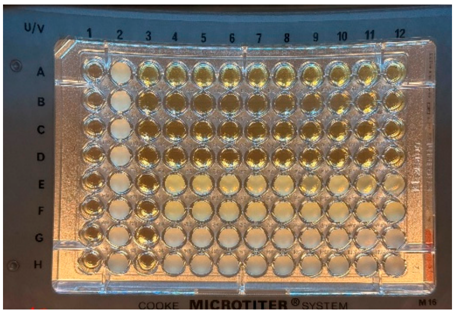

(c)

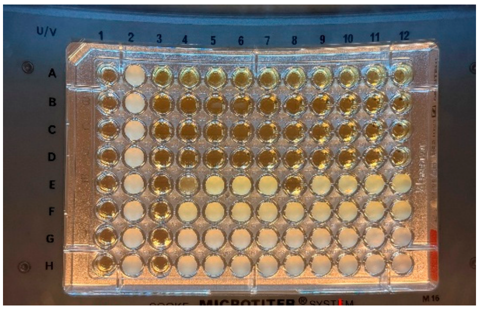

(d)

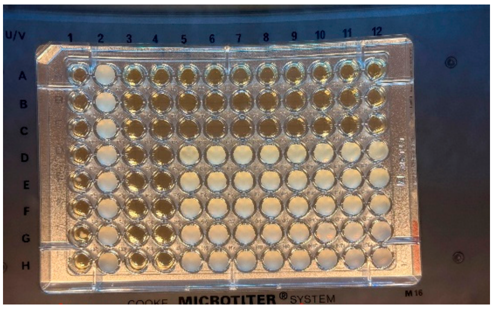

Figure S5. Interaction of CCCP with ceftriaxone on *E. coli* strains

(a) *E. coli* 128673, (b) *E. coli* 132014, (c) *E. coli* 129030, (d) *E. coli* 132009

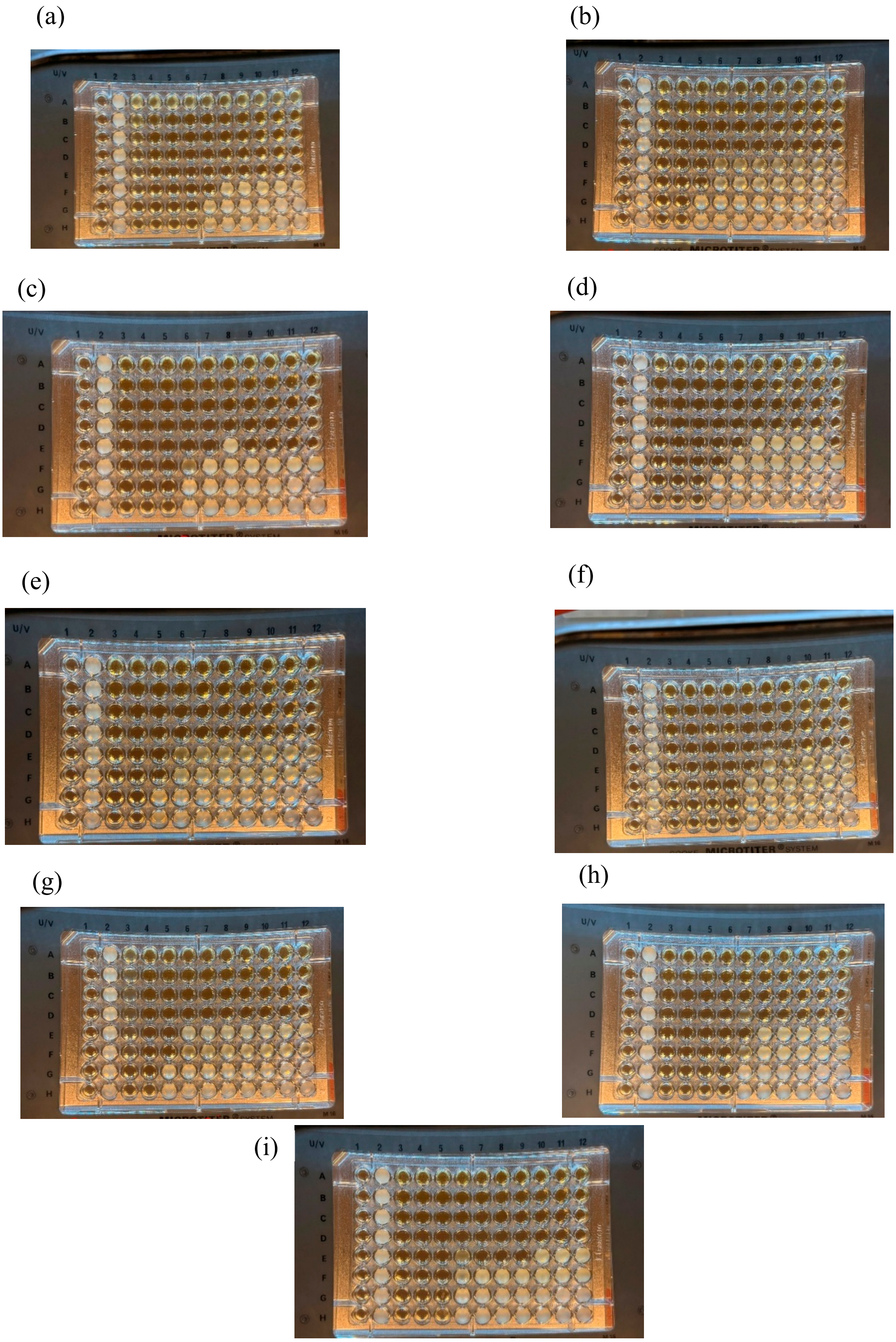

Figure S6. Interaction of CCCP with ciprofloxacin on *E. coli* strains

(a) *E. coli* 131619, (b) *E. coli* 128334, (c) *E. coli* 132009, (d) *E. coli* 128451, (e) *E. coli* 128673, (f) *E. coli* 129351, (g) *E. coli* 131673, (h) *E. coli* 129030, (i) *E. coli* 132014

(a) (b)

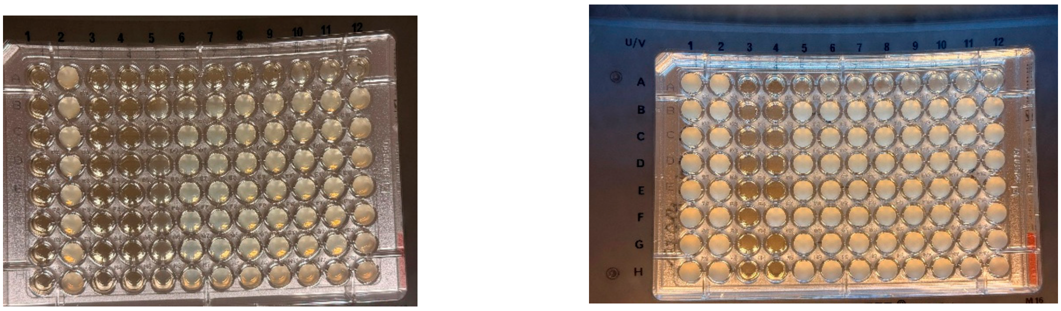

Figure S7. Interaction of TZ with ceftriaxone on *E. coli* strains

(a) *E. coli* 128673, (b) *E. coli* 132014

(a)

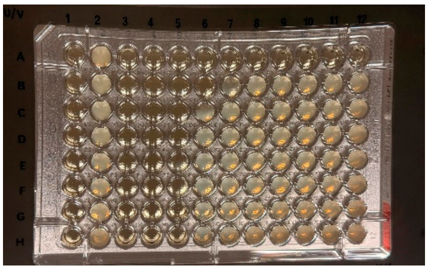

(b)

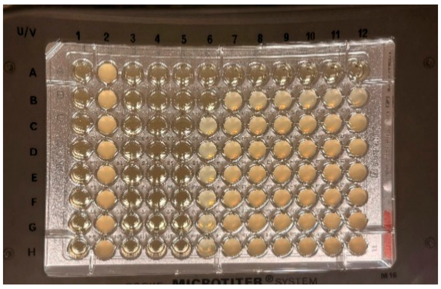

(c)

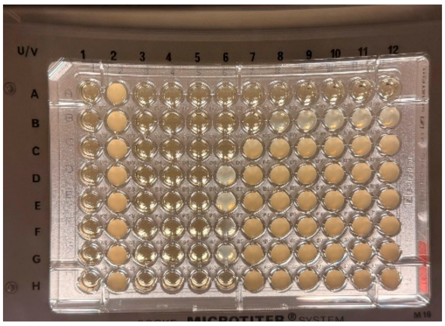

(d)

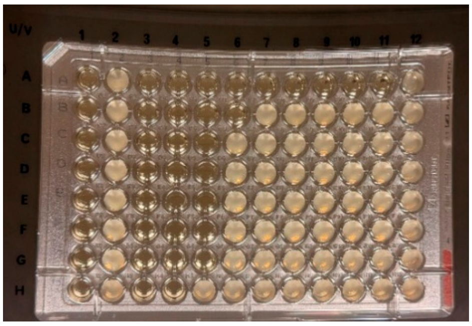

(e)

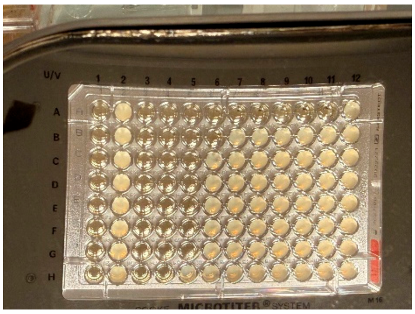

Figure S8. Interaction of TZ with ciprofloxacin on *E. coli* strains

E. coli 128673, (b) *E. coli* 132014, (c) *E. coli* 129030, (d) *E. coli* 128451, (e) *E. coli* 128351

Table S2. Summary table of the starting concentrations used for the investigation of anti-biofilm activity and efflux pump inhibitory effects.

PMZ: promethazine; TZ: thioridazine; CCCP: carbonyl cyanide *m*-chlorophenyl hydrazine; PAβN: phenyl-arginine-β-naphtylamide; DMSO: dimethyl sulfoxide

		PMZ	TZ	CCCP	RES	PAβN
***E. coli* 129351/2022**	**MIC/2**	**50**	**25**	**50**	**100**	**100**
	**MIC/3**	33.33	16.67	33.3	50	50
***E. coli* 132014/2022**	**MIC/2**	50	50	50	100	100
	**MIC/3**	33.33	33.33	33.33	50	50
***E. coli* 129030/2022**	**MIC/2**	50	50	50	100	100
	**MIC/3**	33.33	33.33	33.33	50	50
***E. coli* 128673/2022**	**MIC/2**	100	50	50	100	100
	**MIC/3**	50	33.3	33.3	50	50
***E. coli* 131619/2022**	**MIC/2**	50	100	12	100	100
	**MIC/3**	33.33	50	8.33	50	50
***E. coli* 132009/2022**	**MIC/2**	50	100	50	100	100
	**MIC/3**	33.33	50	33.3	50	50
***E. coli* 128334/2022**	**MIC/2**	50	100	50	100	100
	**MIC/3**	33.3	50	33.3	50	50
***E. coli* 128451/2022**	**MIC/2**	50	50	50	100	100
	**MIC/3**	33.3	33.3	33.3	50	50
***E. coli* 130063/2022**	**MIC/2**	100	100	100	100	100
	**MIC/3**	50	50	50	50	50
***E. coli* 131667/2022**	**MIC/2**	50	100	50	100	100
	**MIC/3**	33.3	50	33.3	50	50

Table S3. Effects of CPFX and PMZ on gene expression (fold change)

CPFX: ciprofloxacin; PMZ: promethazine

	Genes	CPFX	PMZ	CPFX +PMZ
	acrA	1.27	1.18	1.11
	acrB	1.4	1.37	1.24
**Fold change**	*sdiA*	1.23	1.18	0.91
